# Mechanisms of Chemotherapy Resistance in Triple-Negative Breast Cancer—How We Can Rise to the Challenge

**DOI:** 10.3390/cells8090957

**Published:** 2019-08-22

**Authors:** Milica Nedeljković, Ana Damjanović

**Affiliations:** Institute of Oncology and Radiology of Serbia, Pasterova 14, 11000 Belgrade, Serbia

**Keywords:** triple-negative breast cancer, chemoresistance, ABC transporters, cancer stem cells, signaling pathways, hypoxia, apoptosis, receptor tyrosine kinases, microRNA, molecular subtypes

## Abstract

Triple-negative (TNBC) is the most lethal subtype of breast cancer owing to high heterogeneity, aggressive nature, and lack of treatment options. Chemotherapy remains the standard of care for TNBC treatment, but unfortunately, patients frequently develop resistance. Accordingly, in recent years, tremendous effort has been made into elucidating the mechanisms of TNBC chemoresistance with the goal of identifying new molecular targets. It has become evident that the development of TNBC chemoresistance is multifaceted and based on the elaborate interplay of the tumor microenvironment, drug efflux, cancer stem cells, and bulk tumor cells. Alterations of multiple signaling pathways govern these interactions. Moreover, TNBC’s high heterogeneity, highlighted in the existence of several molecular signatures, presents a significant obstacle to successful treatment. In the present, in-depth review, we explore the contribution of key mechanisms to TNBC chemoresistance as well as emerging strategies to overcome them. We discuss novel anti-tumor agents that target the components of these mechanisms and pay special attention to their current clinical development while emphasizing the challenges still ahead of successful TNBC management. The evidence presented in this review outlines the role of crucial pathways in TNBC survival following chemotherapy treatment and highlights the importance of using combinatorial drug strategies and incorporating biomarkers in clinical studies.

## 1. Triple-Negative Breast Cancer

Worldwide, breast cancer is the most commonly diagnosed type of cancer in women and the leading cause of cancer-related death [1]. Triple-negative breast cancer (TNBC) is defined by the lack of expression of estrogen (ER) and progesterone (PR) receptors as well as the absence of human epidermal growth factor receptor 2 (HER-2) overexpression/amplification. TNBC accounts for 10–20% of annually diagnosed breast cancer cases and commonly occurs in younger women, especially of African ancestry [2]. TNBC is poorly differentiated and has a higher proliferative rate compared to hormone receptor-positive (HR+) tumors [3]. It is associated with high recurrence rates, high incidence of distant metastases, and poor overall survival [3]. The pattern of recurrence differs in TNBC compared to HR+ breast cancer. Namely, disease progression and recurrence typically occur within the first 3–5 years after diagnosis while distant metastases present to the brain and lung much more commonly in TNBC [3,4]. Those patients who remain in remission after the first five years have a similar prognosis as patients with HR+ breast cancer.

TNBC is diagnosed by immunohistochemistry (IHC); however, triple-negative phenotype shows significant overlap with the basal-like molecular subtype of breast cancer. In landmark studies based on gene expression profiling, Perou et al. and Sorlie et al. identified five molecular subtypes of breast cancer with distinctive clinical behavior and outcome: Luminal A, Luminal B, Her-2 enriched, Normal-like and Basal-like [5,6]. Basal-like breast cancers are most commonly triple-negative, leading to a misconception that these two terms are synonymous. However, 70–80% of TNBC are basal-like, while about 70% of basal-like tumors are triple-negative [7]. Recently, a TNBC subgroup lacking basal markers was identified. These tumors are enriched for stem cell and epithelial–mesenchymal transition (EMT) markers and belong to the so-called claudin-low molecular subtype [8]. These findings highlight the heterogeneous nature of TNBC.

## 2. TNBC Chemotherapy Basics

Chemotherapy is currently the only systemic treatment option for TNBC, but optimal protocols are yet to be established [9]. Nevertheless, taxane and anthracycline-based regimens represent the mainstay in TNBC therapy, while platinum-based chemotherapy has shown promising results in the neoadjuvant and metastatic settings [9].

Despite the aggressive nature of TNBC, 20% of patients present a pathologic complete response (pCR) after neoadjuvant chemotherapy [10]. However, TNBC patients that did not achieve pCR are several times more likely to suffer an early recurrence and die from metastatic disease compared to HR+. On the whole, TNBC patients have a significantly worse overall survival compared to those suffering from non-TNBC breast tumors despite better pCR rates; a phenomenon termed “triple negative paradox” [11]. The differences in clinical outcomes following neoadjuvant treatment imply that a subset of TNBCs are sensitive to chemotherapy while the majority become resistant during treatment or are intrinsically less susceptible. Both mechanisms are likely present in the tumors.

In this review, we will shed light on major established processes that give rise to chemoresistance in TNBC, and we will especially focus on novel strategies to overcome them. In addition, we will explore TNBC molecular heterogeneity and its therapeutic implications. In doing so, we do not strive to be encyclopedic but will concentrate on recent data, novel therapeutic modalities, and existing controversies.

## 3. TNBC Chemoresistance

### 3.1. ABC Transporters

Chemotherapy resistance presents a significant hurdle for successful cancer treatment, especially in the metastatic setting where it accounts for 90% of therapy failure [12]. Numerous mechanisms can lead to the development of chemoresistance, among which transporter-mediated drug efflux is one of the most thoroughly validated. ATP-binding cassette (ABC) transporters utilize ATP to efflux various compounds across cellular membranes, including a wide range of anti-cancer drugs with different structures and properties [13]. A number of ABC transporters are strongly implicated in chemoresistance of numerous solid tumors, including breast cancer [14]. In particular, multidrug-resistant protein-1 (ABCC1/MRP1), breast cancer resistance protein (ABCG2/BCRP) and multidrug-resistant protein-8 (ABCC11/MRP8) were expressed significantly more, and more frequently in TNBC compared to other breast cancer subtypes [15,16]. The role of ABCC1 in TNBC chemoresistance is further supported by the finding that neoadjuvant chemotherapy increased ABCC1 protein expression in TNBC [17]. Moreover, activation of the hedgehog pathway in TNBC cells led to the acquisition of drug resistance due to the upregulation of ABC transporters [18]. ABCG2 is strongly implicated in chemoresistance of stem cells in TNBC [19]. Consistent with the importance of ABCG2 in TNBC resistance, its downregulation via the inhibition of growth hormone receptor, sensitized TNBC cells to chemotherapy [20]. 

ABCC1 confers resistance to anthracyclines, taxanes, mitoxantrone, methotrexate, and other agents whereas ABCG2 transports drugs such as 5-Fluorouracil, methotrexate, doxorubicin, irinotecan, mitoxantrone, and others [13]. ABCC11 is at the early stages of investigation compared to ABCC1 and ABCG2 but is known to confer resistance to 5-Fluorouracil and methotrexate [21]. It is evident that ABCC1, ABCG2 and ABCC11 have a broad and extensively overlapping substrate specificity. Together they confer resistance to chemotherapy drugs that represent the backbone of current TNBC treatment. 

There are two main approaches in targeting ABC transporters as a means of overcoming chemoresistance: inhibition of their activity and inhibition of their expression. However, the first few generations of ABC transporters activity inhibitors were too toxic to be beneficial, lacked selectivity, or had an insufficient effect on drug accumulation [22]. Despite poor initial results, research in ABC transporter inhibitors is vigorously continuing. Several nonsteroidal anti-inflammatory drugs (NSAIDs) were able to sensitize resistant cell lines overexpressing ABCC1 to cytotoxic drug substrates [23]. NSAID sulindac, in combination with epirubicin, showed preliminary anti-tumor activity in a phase I clinical trial on patients with advanced malignancies, including breast cancer, thus encouraging further investigation [24]. More recently, sulindac, together with docetaxel, was tested in a phase II clinical trial in recurrent or metastatic breast cancer (NCT00039520). However, study results are yet to be formally presented making it difficult to draw conclusions about sulindac clinical value in breast cancer. PZ-39 is an ABCG2 inhibitor with a two-way mode of action [25]. It not only inhibits ABCG2 activity but also accelerates its degradation [25]. Currently, a wide range of natural products are being tested as ABCG2 and ABCC1 activity inhibitors that can be safely combined with chemotherapy due to low toxicity [22]. Interestingly, tyrosine kinase inhibitors (TKIs), while substrates of ABC transporters at lower concentrations, serve as potent inhibitors (especially of ABCG2) at high concentrations [26]. Currently, a number of clinical trials are testing various TKIs (mostly aimed at epidermal growth factor receptor-EGFR and vascular endothelial growth factor receptor-VEGFR) in TNBC, often in combination with chemotherapy [27,28]. However, the ability of TKIs to inhibit ABC transporter activity is generally not taken into account when designing these trials and TKI doses are not modulated accordingly. Since TNBC highly expresses ABCG2, ABCC1 and ABCC11, the implication that TKIs could not only work against their primary targets, but also enhance the effects of chemotherapy in combination treatment, needs to be thoroughly explored. Nanoparticle drug delivering systems also showed promise in inhibiting ABCC1- and ABCG2-based chemoresistance. For instance, the redox-responsive polymeric micelles containing indomethacin (an NSAID) as a chemosensitizing agent and carrying paclitaxe, strongly inhibited the growth of breast cancer cells [29].

A novel approach in attenuating ABC transporter-mediated chemoresistance centers on the use of small interfering RNA (siRNA) and microRNA to downregulate their expression. siRNA can precisely and effectively silence targeted genes [30]. This approach gained traction especially after anti-tumor potential of RNA interference (RNAi)-based therapeutics was demonstrated in cancer patients [30]. Several groups used RNAi to block ABCG2 [31,32] and ABCC1 [33] protein expression in resistant cell cultures, thus restoring the therapeutic benefits of cytotoxic drugs that are their substrates. A number of microRNAs demonstrated inhibitory effects on ABCG2 expression in breast cancer cells [34].

Inhibiting a single transporter may not be enough to overturn chemoresistance in vivo due to high redundancy in transport function. However, inhibiting several transporters is burdened with high toxicity. This issue is entirely circumvented with a novel strategy based on agents that are poor substrates of ABC transporters. One group recently reported that FL118, a semisynthetic analog of camptothecin, is a poor substrate of ABCG2 and ABCB1 [35,36]. FL118 had a stronger negative effect on tumor growth compared to irinotecan in xenograft models that highly expressed ABCG2 [35]. In addition, cell cultures with high expression of ABCG2 were still sensitive to FL118 [35]. 

Clinical data regarding ABC transporter inhibitors in breast cancer is limited. However, preclinical information presented in this review is supportive of the role of ABC transporters in TNBC chemoresistance. These tumors might be well suited for the use of ABC transporter inhibitors.

### 3.2. Cancer Stem Cells

**Figure 1:**
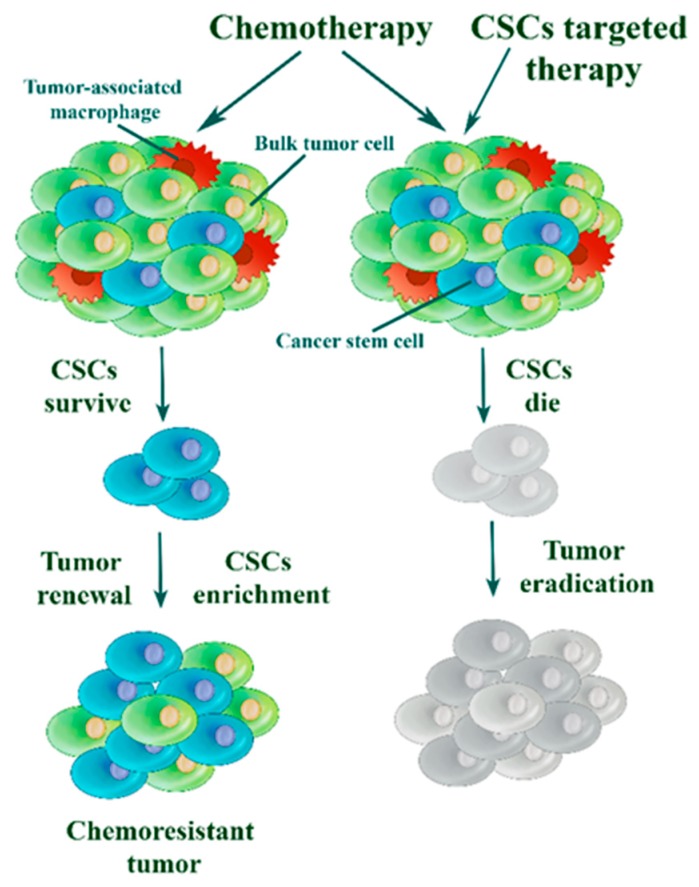
Cancer stem cell theory. Chemotherapy reduces bulk tumor burden but resistant cancer stem cells (CSCs) survive and are able to drive tumor recurrence. Specifically targeting CSCs is necessary to achieve stable tumor remission.

It is well documented that in solid tumors, there exists a subpopulation of cells with a unique aptitude for tumor renewal [37]. These cells are named cancer stem cells (CSC). CSCs have been implicated in tumorigenesis, tumor heterogeneity, recurrence, and metastasis [37,38]. They have self-renewal properties and an ability to re-establish a tumor following treatment (Figure 1).

Specifically, in breast cancer, a substantial increase of CSC presence was noted in the residual tumors following exposure to conventional chemotherapy [39]. This finding implies that breast CSCs are resistant to treatment with their selective survival resulting in a residual tumor enriched with tumor-initiating cells. Subsequent studies also detected a higher percentage of CSCs in primary breast tumors following neoadjuvant chemotherapy, thereby confirming the initial result [40]. This phenomenon is of particular importance in TNBC, which has very limited therapy options and seems to be intrinsically enriched in CSCs compared to luminal and HER2+ breast cancer subtypes [41,42]. Many lines of evidence support the notion of CSC importance in TNBC, such as the positive correlation observed between the expression of stem cell markers (CD44, ALDH1) and lower survival rates of TNBC patients [43,44]. Accumulating data suggests that chemoresistant CSCs may be a dominant factor in TNBC relapse. RNA transcripts of CSCs associated genes were upregulated in TNBC biopsies following chemotherapy [45]. Treatment of TNBC cell lines with gemcitabine or paclitaxel promoted the expression and activity of hypoxia-inducible factors (HIFs) which resulted in the increase of CSC population signaling and the upregulation of ABCB1 expression [46]. Inhibition of factors crucial for CSC maintenance, sensitized TNBC cells to cytotoxic drugs [47]. 

Mechanisms behind CSC ability to evade chemotherapy are still unclear. It is well established that CSCs are relatively quiescent when compared to more differentiated cells. This dormant behavior of CSCs could provide them with a natural defense against cytotoxic agents that are generally most effective against fast-dividing cells [37]. Secondly, CSCs have highly expressed ABC transporters, most notably ABCG2, which confer resistance to a broad spectrum of cytotoxic agents. Several studies have reported ABCG2 as an important marker of the so-called, side population of CSC [48]. In addition, Britton et al. demonstrated that breast cancer side population cells had increased ABCG2 expression, higher resistance to mitoxantrone, and were associated with TNBC subtype [19].

Therapies that could target CSCs are generating great interest, although much more work remains to be done. ABCG2 inhibition could represent a chief strategy in this setting, contributing to the eradication of tumor-initiating CSCs. A proof of concept was provided by a novel ABCG2 inhibitor, YHO-13351, which sensitized side population CSCs to irinotecan in vitro and in vivo [49]. 

The second approach includes targeting CSC surface antigens. Nanoparticles coated with hyaluronic acid (a CD44 ligand) and carrying chemotherapy agents, were able to target breast CSCs, both in vitro and in vivo while sparing healthy cells [50]. Various other CD44 targeting nanocarriers are currently being investigated. They serve as delivery vehicles for a number of therapeutic agents, including cytotoxic drugs and siRNAs [51]. 

**Figure 2:**
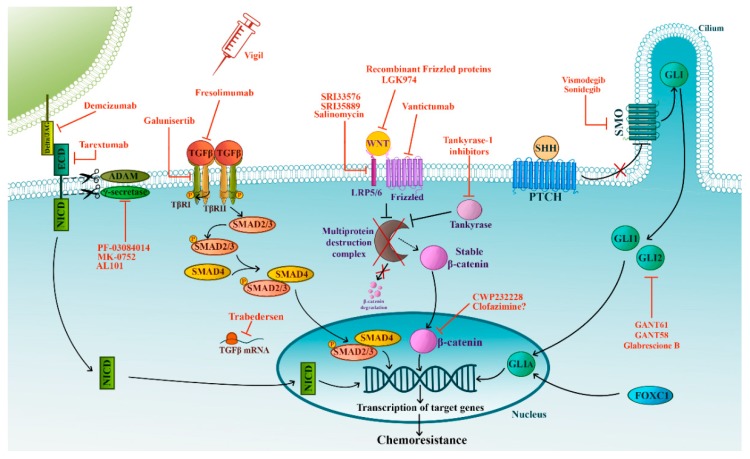
Overview of developmental pathways and their potential inhibitors in triple-negative breast cancer (TNBC). TGF-β (transforming growth factor-beta), Notch, Wnt/β-catenin and hedgehog (Hh) pathways all have crucial roles in initiation, progression, CSC maintenance, metastasis, and chemoresistance of TNBC. A number of inhibitors have recently been developed to target these pathways. Inhibitors are shown in red.

Targeting signaling pathways crucial for CSC self-renewal represents a promising third approach. TGF-β (transforming growth factor-beta), Notch, Wnt (wingless)/β-catenin, Hh (Hedgehog) developmental pathways all have essential roles in CSCs. These pathways are discussed in greater detail below (Figure 2).

#### 3.2.1. TGF-β Pathway

TGF-β is a member of a large cytokine superfamily that consists of over 30 related growth factors, including three TGF-β isoforms (TGF-β1–3) [52]. The pathway is engaged when TGF-β binds to type II TGF-β receptor (TGF-βR), which subsequently recruits and transphosphorylates the type I TGF-βR forming a receptor complex. The now active type I TGF-βR, in turn, recruits and phosphorylates the main effectors of this pathway—Smad2 and Smad3. Phosphorylated Smad2/3 interact with Smad4 forming a heteromeric complex that is transported into the nucleus where it regulates the expression of numerous target genes [52].

In oncology, TGF-β signaling is known to promote EMT, proliferation, angiogenesis, metastatic spread, chemotherapy resistance, and has an immuno-modulating effect [53]. Moreover, the TGF-β pathway is critical for the regulation of breast CSCs [54]. Namely, human breast cancer cell lines exposed to TGF-β underwent EMT and acquired CSC properties, including chemoresistance [54]. Chemotherapy treatment of TNBC was revealed to increase TGF-β signaling [45]. In addition, the use of a TGF-βR inhibitor in TNBC xenografts prevented the re-establishment of tumors following chemotherapy [45]. In concordance with these findings, a recent study showed that both TGF-β expression and breast CSC markers were increased in epirubicin resistant TNBC cell lines [55]. Together, these results imply a vital role of TGF-β signaling in the acquisition of stemness and TNBC chemotherapy resistance, thus providing a rationale for new therapeutic strategies. Targeting the TGF-β pathway mostly focuses on small molecule inhibitors aimed at TGF-βR. Several of these are currently undergoing clinical evaluation. For instance, an ongoing phase I clinical trial is investigating galunisertib (TKI with low toxicity) in combination with chemotherapy in metastatic TNBC and is expected to conclude in 2021 (NCT02672475). The second approach centers on the anti-TGF-β monoclonal antibodies (mAbs). Fresolimumab was evaluated in a clinical trial in metastatic breast cancer but generated disappointing results (NCT01401062). Other strategies, like advanced vaccines (vigil) and antisense oligonucleotides (trabedersen), are still at relatively early stages of investigation and have, so far, generated mixed results [56,57].

Several challenges remain ahead of TGF-β-based TNBC therapy, including selectivity/specificity issues of TGF-βR inhibitors and the accessibility of the TGF-β to mAbs [58]. Additionally, the TGF-β pathway acts as a tumor suppressor in early-stage cancers, including breast cancer [52]. Therefore, the implementation of inhibitors will need to be extremely careful in order to suppress the tumorigenic arm of the pathway while encouraging the tumor-suppressive one.

#### 3.2.2. Notch Pathway

The canonical Notch signaling pathway is comprised of four cell surface receptors (NOTCH 1–4) and five transmembrane ligands (Delta-like 1,3,4 and JAGGED-1,2). Cell-to-cell contact is required for Notch activation. Binding of ligands on neighboring cells induces successive cleavages by ADAM proteases and γ-secretase resulting in the release of the intracellular domain (NICD) of the receptor. NCID translocates to the nucleus where it initiates transcription of numerous target genes [59]. 

Altered Notch signaling has diverse effects in human tumors and is implicated in all of the hallmarks of cancer, from immune system evasion to maintenance of CSCs [59]. Notch 1–4 signaling is established as crucial for the maintenance of breast CSCs and highly correlates with resistance to chemotherapy [60,61]. Specifically, in TNBC, Notch-1/3/4 overexpression/amplification was detected and associated with the induction of proliferation, invasiveness, and tumorigenesis [62,63,64]. Recently, constitutive Notch-3 signaling was suggested as a driver of oncogenic program in the basal subset of TNBC [65]. However, Zhang et al. showed that Notch-3 could also act as a tumor suppressor in TNBC cell lines where it inhibited EMT [66]. Doxorubicin induced Notch-1 signaling in breast cancer cell lines, which led to increased ABCC1 expression. Importantly, γ-secretase inhibitor (GSI) reverted the Notch-1 induced upregulation of ABCC1, rendering the cells more susceptible to doxorubicin [67]. This effect was also confirmed in TNBC cells, where GSI enhanced the efficacy of doxorubicin [68]. In a similar vein, siRNA-mediated knock-down of Notch-1 inhibited the growth of TNBC cell lines and increased their sensitivity to docetaxel and doxorubicin [64]. In concordance with these findings, Notch-1 inhibitors had a synergic effect with docetaxel in TNBC and showed potent anti-tumor action in breast CSCs and patient-derived xenograft models [69]. 

Presented preclinical data provide evidence of Notch signaling as crucial for TNBC chemoresistance and demonstrates the ability of Notch inhibitors to sensitizes cells, including CSCs, to cytotoxic agents. Therefore, it seems logical to deploy GSIs concurrently with chemotherapy for treating TNBC patients. This strategy was investigated in two recent phase I clinical studies of GSIs in advanced breast cancer, including TNBC [70,71]. PF-03084014 GSI, in combination with docetaxel, was well tolerated and showed clinical benefit in patients with advanced TNBC [70]. Another GSI, MK-0752, together with docetaxel, had limited anti-tumor activity but importantly, reduced breast CSC burden in a number of patients after multiple treatment cycles [71]. However, in a recent preclinical study, AL101 (a novel GSI), demonstrated significant anti-tumor effects in TNBC patient-derived xenografts that displayed abnormal Notch signaling [72]. Moreover, a phase II clinical trial is currently investigating AL101 as monotherapy in adenoid cystic carcinoma (NCT03691207). This implies that GSIs could have efficacy in TNBC treatment even as monotherapy. While γ-secretase is the most thoroughly investigated target, other approaches to inhibit Notch signaling are also being evaluated in clinical studies. Tarextumab, a first in class anti-Notch 2/3 antibody, recently completed a phase I clinical trial in advanced solid tumors, including breast cancer with acceptable levels of toxicity [73]. Preliminary activity against Notch signaling was observed [73]. Demcizumab, an anti-DLL4 mAb, completed several clinical trials in solid tumors as a single agent or in combination with chemotherapy. However, after demcizumab failed to provide any clinical benefit in two recent phase II trials (NCT02259582, NCT02289898), further development was discontinued. These results illustrate the challenge of developing novel anti-cancer agents but do not lessen the therapeutic value of targeting Notch signaling in TNBC.

#### 3.2.3. Wnt/β-Catenin Pathway

Wnt signaling is associated with tumor initiation, stemness, and metastatic spread [74]. In the absence of Wnt, β-catenin is rapidly degraded due to the action of the multi-protein destruction complex. Binding of Wnt to its receptors and co-receptors (Frizzled and low-density lipoprotein receptor-related proteins (LRP5/6), respectively) ultimately causes the dissolution of the destruction complex stabilizing β-catenin. Accumulating β-catenin is then free to translocate to the nucleus and activate the transcription of Wnt targeted genes [74]. In addition to this canonical pathway, two β-catenin-independent pathways also exist: planar cell polarity pathway (PCP) and calcium-dependent pathway, both of which regulate the cytoskeleton and are crucial for cancer cell migration [75].

Extensive literature data highlights the key role of deregulated Wnt/β-catenin signaling in TNBC and its association with aggressive tumor phenotype and poor outcome. TNBC patients with aberrant Wnt/β-catenin signaling are more likely to develop distant metastases, especially to the brain and lung [76]. Wnt signaling was even suggested as necessary for TNBC development [77]. Namely, TNBC cells with knocked-down β-catenin had remarkedly slower growth, impaired migration ability, were more susceptible to chemotherapy and formed significantly smaller tumors in murine models [77]. In addition, the same study connected Wnt/β-catenin signaling with TNBC stemness as its knock-down also reduced the stem cell population [77]. Building on these results, β-catenin was demonstrated to have a synergistic effect with Nek2B on chemotherapy resistance in TNBC [78]. Other constituents of the Wnt/β-catenin pathway are also upregulated in TNBC. FZD6 (a member of the Frizzled receptor family) was overexpressed in TNBC and linked with adverse clinico-pathological tumor properties [79]. FZD6, acting as a component of the non-canonical Wnt pathway, had a crucial role in cellular motility, invasion, and metastasis in TNBC [79]. FZD8-mediated Wnt signaling was shown to have a major part in TNBC chemoresistance as it was significantly enhanced in residual cells following neoadjuvant chemotherapy [80]. Paralleling these findings, LRP6 overexpression was demonstrated in TNBC, and its knock-down not only inhibited cell proliferation but had an even stronger negative effect on cell migration and invasion [81]. The crucial role of Wnt/β-catenin signaling in TNBC tumorigenesis is doubtless and, as such, represents a logical therapeutic target. Numerous preclinical studies have attested to the merit of using Wnt/β-catenin inhibiting agents. For instance, novel benzimidazole compounds, SRI33576 and SRI35889 produced pro-apoptotic effects in TNBC cell lines by downregulating LRP6 and therefore inhibiting Wnt/β-catenin signaling [82]. Treatment with salinomycin sharply reduced β-catenin signaling and was able to suppress breast CSC proliferation, invasion, and self-renewal while inducing apoptosis [83]. CWP232228, a small molecule that selectively inhibits Wnt pathway signaling by blocking nuclear β-catenin interaction with T-cell factor, reduced tumor growth in TNBC xenograft models and had a strong effect against chemoresistant breast CSC both in vitro and in vivo [84]. A repurposed drug clofazimine demonstrated efficacy in reducing the proliferation of TNBC cells and tumor growth in xenograft models [85]. Clofazimine negatively impacted β-catenin accumulation in the cytosol, thus downregulating the pathway and reducing the expression of ABC transporters [85]. Moreover, it showed a marked synergistic effect with doxorubicin with an excellent toxicity profile [85]. Combined inhibition of tankyrase-1, which antagonizes the destruction complex, and polo-like kinase 1 dramatically reduced TNBC cells invasiveness and survival [86]. A novel recombinant human Frizzled-7 protein antagonist repressed proliferation, invasion, and angiogenesis while sensitizing TNBC cells to docetaxel both in vivo and in vitro [87]. This wealth of preliminary data has culminated in the clinical investigation of a number of agents that target Wnt/β-catenin signaling at different levels. LGK974, a small molecule that blocks Wnt ligand secretion, is currently being tested in patients with Wnt-ligand dependent malignancies, including TNBC (NCT01351103). Another phase I trial tested vantictumab, an antibody that blocks several Frizzled receptors, together with paclitaxel in advanced or metastatic breast cancer (NCT01973309). However, formal results have not yet been made available. PTK7-ADC, an antibody–drug conjugate targeting a component of the Wnt/β pathway, is currently undergoing evaluation in metastatic TNBC as a combination therapeutic (NCT03243331). 

In the last decade, great strides have been made in the understanding of the structure and interaction of Wnt/β-catenin signaling pathway constituents. Multiple Wnt/β-catenin targeted inhibitors were designed in the wake of this knowledge. These inhibitors have shown efficacy in TNBC both as monotherapy and as sensitizing agents, although the research is still in early clinical phases. Wnt/β-catenin pathway is not fully elucidated as of yet and its continued characterization will certainly provide opportunities for the development of future anti-tumor agents.

#### 3.2.4. Hedgehog Pathway

The Hh signaling pathway is an elaborate network critical for embryonic development and tissue regeneration. Altered signaling of this pathway has been implicated in stem cell renewal and carcinogenesis [88]. In essence, the Hh pathway consists of three secreted ligands, of which the Sonic Hedgehog (SHH) is the most broadly expressed, and transmembrane receptor/co-receptors Patched (PTCH) and Smoothened (SMO). Three glioma-associated oncogene transcription factors (GLI1–3) are the main effectors and regulate the expression of many target genes, such as ABCG2 and VEGF [89]. The canonical pathway is activated when SHH binds PTCH destabilizing it and thus alleviating its repression of SMO. Activated SMO allows the formation of the full-length activator form of GLI transcription factors—GLIA. Upon translocation to the nucleus, GLIAs upregulate target genes.

GLI1/2 are linked to cell survival, proliferation, invasion, EMT, angiogenesis, and chemoresistance in various human tumors [88]. Growing evidence connects Hh signaling with more aggressive clinical behavior of TNBC. Overexpressed SHH stimulated the migration, invasion, and proliferation of TNBC cells in vitro while enhancing lung dissemination in vivo [89]. Similarly, increased expression of GLI1 promoted the survival, migration, invasion, and metastasis of TNBC cells [90]. In concordance with these findings, the inhibition of the Hh pathway reduced motility and self-renewal capacity of TNBC cells but promoted apoptosis [90]. Mechanically, Hh signaling increases TNBC growth, invasiveness, and metastatic dissemination by enhancing the expression of extracellular matrix remodeling metalloproteases and VEGFR, thus stimulating angiogenesis [90,91]. Accumulating evidence suggests frequent non-canonical Hh pathway activation in TNBC due to the action of several other oncogenic pathways [92]. For instance, Han et al. reported the activation of GLI2 in a SMO independent manner [93]. Hh signaling is also strongly linked with CSC in TNBC. Both GLI1 and GLI2 are upregulated in breast CSCs, while the induction of cell differentiation considerably reduced their expression [94,95]. GLI1 seems to be crucial, even required, for EMT in breast cancer [96]. Several studies correlated Hh activity status with TNBC histopathologic characteristics and patient outcomes. Hh signaling was found to be associated with larger tumor size, high grade, high stage, and with poor prognosis in TNBC [97,98]. Moreover, Hh signaling is implicated in chemoresistance of breast tumors. Treatment of breast cancer cell lines with docetaxel caused the activation of Hh signaling leading to the survival and expansion of breast CSC [95]. GLI1 was overactivated via non-canonical pathway following exposure of malignant cells to cytotoxic drugs and, subsequently, stimulated the expression of ABC transporters [18].

Extensive preclinical information in regard to Hh signaling has stimulated numerous investigations of this pathway as a therapeutic target. By far the majority of inhibitors are directed against SMO, the most druggable member of the pathway. While these agents showed good preclinical performance and a few have been approved for the treatment of other tumors, their efficacy in breast cancer was disappointing. Namely, preliminary data from clinical trials investigating SMO inhibitors in breast cancer, including TNBC, showed only limited benefit and a number were terminated early (NCT02027376, NCT01071564, NCT01576666, NCT01757327). However, vismodegib, an SMO inhibitor approved for the treatment of basal-cell carcinoma, is currently undergoing evaluation in TNBC patients (NCT02694224). SMO independent activation of the Hh pathway was demonstrated in TNBC and could partially account for the lack of efficacy of SMO inhibitors [18,92]. Preclinical data implies that the use of GLI inhibitors, although technically more difficult, might be preferred for TNBC treatment. These compounds inhibit GLI function by varied mechanisms and are classified as direct (such as GANT61, GANT58, Glabrescione B) or indirect inhibitors. Of the direct GLI inhibitors, GANT61 seems the most promising. It showed strong action in TNBC cell lines where it stimulated apoptosis, reduced proliferation, and decreased CSC population even when SMO inhibitors proved ineffective [93,99]. However, none of the GLI inhibitors have entered clinical trials as of yet. 

Taking all data into account, the crucial role of developmental pathways in TNBC initiation, progression, CSC maintenance, metastasis, and chemoresistance is undeniable. Based on the evidence presented in this review, it would seem that TNBC patients could benefit greatly from the development of new therapeutic agents targeting developmental pathways. However, significant challenges remain if these drugs are to be brought into the clinic. High toxicity is a concern since any systemic treatment with such inhibitory agents would undoubtedly impact normal stem cells and thus have detrimental effects on adult tissue homeostasis and repair. Moreover, while we have, in the interest of clarity and limited space, presented these pathways independently, considerable cross-talk and collaboration exists between them. This signaling network, which is yet to be fully elucidated, would need to be addressed if successful treatment is to be achieved.

### 3.3. Hypoxia

**Figure 3:**
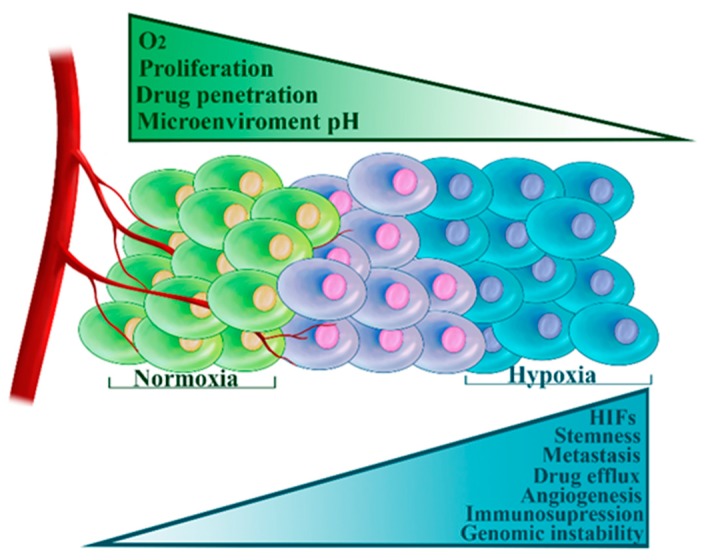
Role of hypoxia in tumor chemoresistance. Hypoxia induces numerous mechanisms that interfere with cytotoxic drugs and hinder their success.

Hypoxia is a term that refers to insufficient tissue oxygen supply. As the tumor expands, blood vessels grow haphazardly, often being cut-off or destroyed [100]. Acute hypoxia develops due to a transient lack of oxygen, while chronic hypoxia arises due to increased diffusion distances—malignant cells are too far from the blood vessel to receive adequate oxygen [100]. Low oxygen levels stabilize HIFs, which in turn, regulate transcriptional activation of a large cluster of genes allowing the cells to survive in these harsh conditions [100]. Hypoxia is a fundamental characteristic of the tumor microenvironment and is associated with tumor aggressiveness, metastatic potential, and resistance to therapy [100]. Hypoxia contributes to chemoresistance in several essential ways (Figure 3). Firstly, insufficient vasculature hinders drug penetration [100]. Secondly, hypoxia leads to the acidic tumor microenvironment, which compromises the uptake of certain drugs widely used in TNBC treatment [101]. Thirdly, cytotoxic effects of a number of drugs are oxygen dependent [102]. Fourthly, hypoxia induces the breast CSC phenotype [103]. Fifthly, hypoxia directly or indirectly modulates tumor immunity by activating immunosuppressive signaling pathways and acting as a barrier to immune effector cells [104]. Finally, hypoxia stimulates cellular adaptations that act as obstacles to successful treatment. These include: increased expression of ABC transporters (including ABCG2 and ABCC1) [105,106]; decreased proliferation [102]; complex modulation of cellular senescence and apoptosis [102]; induction of autophagy that aids in tumor survival [107]; enhanced genetic instability and subsequent clonal selection of aggressive phenotypes [102]; upregulation of pro-angiogenic factors [102] and repression of E-cadherin thus promoting metastatic spread [102]. 

TNBC frequently shows morphological features that are characteristic of hypoxia, such as the presence of fibrotic and necrotic areas [108]. Another evidence for the importance of hypoxia in these tumors comes from the study by Tam et al. who demonstrated that the expression of carbonic anhydrase IX (CAIX), a key HIF-1 regulated gene, was associated with TNBC subtype and shorter survival [109]. More directly, several studies have shown the hyperactivity of HIF-1 in TNBC and its association with poor survival [110,111]. Hypoxia, via HIF-1, also promoted EMT transition and induced the invasion of TNBC cells [96]. A recent publication has even suggested HIF-1 and hypoxia as hallmarks of TNBC [111,112]. Adding to these findings, it was recently demonstrated that HIF-1 expression, together with CAIX, has an unfavorable effect on TNBC patients’ survival [113]. In a similar vein, the metabolic silencing of HIF-1 in TNBC xenografts markedly reduced tumor growth rates [114].

Hypoxia has many profound effects on malignant cells rendering the tumors more challenging to treat. However, since hypoxia is mainly a characteristic of solid cancers, it also represents an opportunity to target tumor tissues. There are two main strategies in exploiting hypoxia: the use of hypoxic cytotoxins and inhibition of molecular targets that allow cellular survival in low oxygen condition [115]. Hypoxic cytotoxins come in the form of hypoxia-activated prodrugs (HAPs). The prodrug is nontoxic but is converted to a cytotoxic free radical by the action of intracellular one-electron reductases. In conditions of normoxia, the free electron from the radical is immediately transferred to molecular oxygen, thereby recreating the harmless prodrug [115]. Based on chemical structure, HAPs can be classified into several groups: nitro groups, aliphatic and aromatic N-oxides, quinones, and transition metals [115]. For example, TH-302 showed promising results in a phase II trial in pancreatic cancer [116]. Apaziquone and TH-4000 (a hypoxia-activated EGFR TKI) are also undergoing assessment in clinical trials [117]. However, significant challenges remain ahead of the successful implementation of HAPs in tumor treatment, including normal tissue toxicity, insufficient delivery, short therapeutic windows due to hypoxia fluctuations within tumors and others. 

The inhibition of molecular targets critical for hypoxia processes represents a promising alternative strategy that could compensate for HAP deficiencies. The main targets of this approach are the HIF family of transcription factors as well as their partners and downstream targets. Several HIF inhibitors have shown high efficacy in preclinical studies. For instance, IDF-11774 reduced HIF-1α accumulation in hypoxic conditions and negatively impacted the growth of colorectal carcinoma both in vitro and in vivo [118]. Since the discovery of HIFs, many inhibitors with diverse modes of action have been identified, but none have progressed to clinical use [119]. The vast majority are indirect inhibitors. Direct, specific agents that inhibit the expression of HIFs or their activity have emerged only recently and are now undergoing clinical trials. PT2385, a novel HIF-2α antagonist, showed encouraging results in a phase I trial on previously treated advanced clear cell renal cell carcinoma [120]. Clinical benefit was reported in more than 2/3 of patients and was accompanied by an acceptable toxicity profile [120]. PT2977, a more potent second-generation HIF-2α antagonist is a subject of several ongoing investigations in solid tumors (NCT02974738, NCT03634540, NCT03401788). 

We hypothesize that direct and selective HIF inhibitors are more appropriate for usage in anti-cancer treatment and will be the focus of future hypoxia-based therapeutic strategies. Literature data regarding the effects of hypoxia in TNBC implies these agents could be important pieces in our arsenal against this disease.

### 3.4. Avoidance of Apoptosis

The complex apoptotic machinery is universally dysregulated in cancer. Evasion of apoptosis is a major hallmark of cancer and has been linked with resistance to various cytotoxic agents critical for the treatment of TNBC, such as paclitaxel, doxorubicin, and cyclophosphamide [121,122]. The importance of apoptotic malfunction in the TNBC prognosis is well documented. For instance, protein expression of pro-survival factors, such as Bcl-2 and Mcl-1, was reported as an indicator of unfavorable outcome in TNBC [123,124]. Moreover, the molecular profiling of residual triple-negative tumors resistant to chemotherapy revealed that *MCL1* is the second most frequently altered gene [125]. There are reports linking MCL-1 expression with chemoresistance [126]. Since MCL-1 was found to be crucial in breast cancer development, its expression likely contributes to intrinsic TNBC chemoresistance [123]. Preclinical studies also demonstrated that Bcl-2 inhibitors, like ABT-199, sensitize TNBC cells to doxorubicin [122]. 

**Figure 4:**
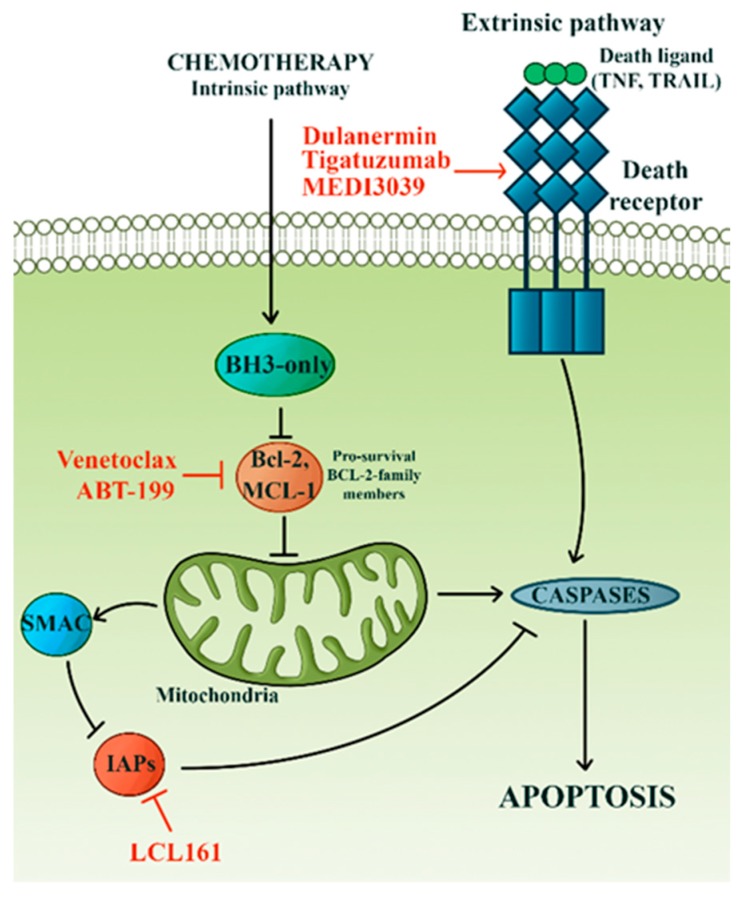
Targeting apoptotic pathways in TNBC. Cancer cells contain two pathways that can trigger apoptosis: intrinsic, that is activated in response to cellular damage, and extrinsic, which is mediated by death receptor activation. Both are potential targets for TNBC treatment.

Targeting deregulated apoptosis represents an attractive approach to cancer therapy (Figure 4). Most studies investigating anti-cancer strategies have focused on Bcl2 family members, TRAIL receptors, and inhibitors of apoptosis (IAPs) [127]. BH3-only proteins are members of Bcl2 family essential for the initiation of apoptosis [128]. Advances in the understanding of BH3-only proteins structure and function have allowed the development of BH3 mimetics—anti-cancer agents that imitate the action of BH3-only proteins, thus promoting apoptosis [128]. Venetoclax, a BH3 mimetic, is currently being tested in several phase I/II clinical trials in advanced breast cancer (NCT03878524, NCT03900884, NCT03584009). Dulanermin, a soluble recombinant human TRAIL, and several death receptor (DR) agonistic mAbs were previously evaluated in clinical trials mostly on advanced solid tumors [129]. However, while these agents were well tolerated, they showed little efficacy and largely failed to improve patient outcomes [129]. For instance, a phase II clinical study investigating tigatuzumab combined with chemotherapy in metastatic TNBC recently concluded with unsatisfactory results (NCT01307891). Further development of tigatuzumab was terminated. The assumption is that current DR agnostic mAbs are unable to trigger a strong enough response in tumor cells. Multivalent DR agonists might represent the solution to this problem. MEDI3039, a highly potent novel multivalent agonist, demonstrated strong anti-tumor efficacy both in-vitro and in-murine models of TNBC [130]. Still, the true potential of this compound can only be revealed in clinical studies. Upon receiving pro-apoptotic stimuli, mitochondria release the second mitochondria-derived activator of caspases (SMAC) which acts as an antagonist of IAPs [131]. This mechanism has inspired the creation of SMAC mimetics as pro-apoptotic, anti-cancer agents [131]. There is evidence that SMAC mimetics could be particularly effective in TNBC [131]. For example, Debio 1143 showed good preclinical performance and is currently undergoing testing in several clinical trials on advanced solid tumors, including TNBC (NCT03270176, NCT01078649, NCT02022098, NCT01930292). Another SMAC mimetic, LCL161, demonstrated high efficacy as a neoadjuvant agent in combination with paclitaxel [132]. In preclinical studies, LCL161 was shown to promote apoptosis and have synergistic effects with paclitaxel [132]. In this phase II clinical trial in localized TNBC, LCL161/paclitaxel combination more than doubled the pCR rate compared to paclitaxel alone, although accompanied by increased toxicity [132]. However, the pCR effect was only present in the TNBC group preselected for the tumor necrosis factor (TNF) gene expression profile [132]. These results do not just highlight the value of LCL161 but demonstrate the crucial role of biomarkers in defining patient populations most likely to respond favorably to treatment.

### 3.5. Role of Signaling Pathways in TNBC Chemoresistance

**Figure 5:**
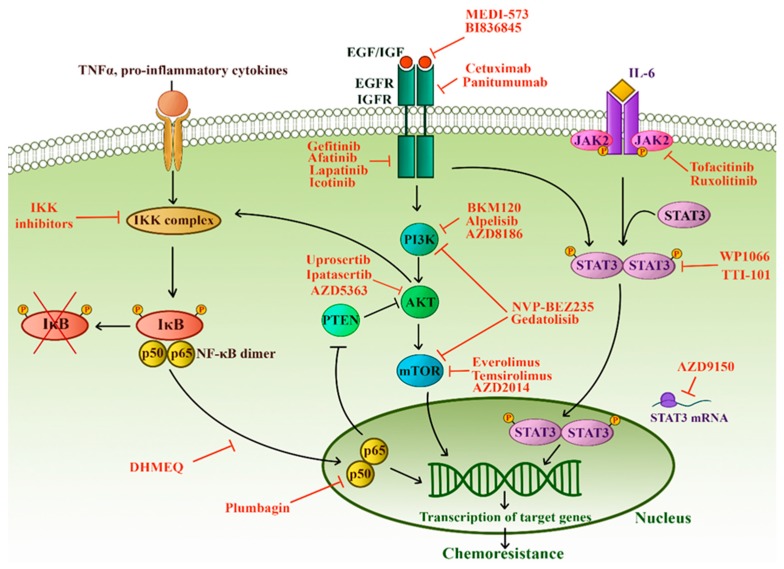
Targeting the nuclear factor kappa-light-chain-enhancer of activated B cells (NF-κB); PI3K-AKT-mTOR (PAM); Janus kinase (JAK)/signal transducer and activator of transcription (STAT) pathways and receptor tyrosine kinases in TNBC. Select inhibitors are shown in red.

An intricate network of signaling pathways governs the survival, growth, and invasion of TNBC. Especially NF-κB; PTEN/PI3K/AKT/mTOR; JAK/STAT and receptor tyrosine kinases are implicated in TNBC chemoresistance and progression. Immense effort has been made into understanding the alterations of these pathways in TNBC, and it is now bearing fruit in the form of targeted therapeutics (Figure 5).

#### 3.5.1. NF-κB

NF-κB (nuclear factor kappa-light-chain-enhancer of activated B cells) family is comprised of five members with the ability to form hetero- and homodimers [133]. When the pathway is not engaged, the dimers are kept inactive due to the binding of inhibitors (IκB). The activation of the NF-κB canonical signaling causes the formation of an active IκB kinase (IKK) complex, which phosphorylates IκB resulting in the release of NF-κB dimers. NF-κB can now freely enter the nucleus and induce the transcription of many target genes [133]. 

NF-κB signaling pathway is a crucial regulator of TNBC—it inhibits apoptosis, controls inflammatory response and angiogenesis, is associated with TNBC progression and poor prognosis [134,135]. Moreover, the level of NF-κB expression in TNBC is several times higher compared to normal breast tissue [134]. It is well established that NF-κB activation mediates chemoresistance in breast and other types of cancer [133]. NF-κB signaling is also upregulated by hypoxia, which, as we have already discussed, has a clear connection with chemoresistance [136].

Since NF-κB has a vital role in many cellular processes, inhibitors of this pathway have been generating great attention. More than 750 inhibitors have been described so far, including small molecules, peptides, siRNAs, antioxidants, microbial, and viral proteins [137]. The majority of these agents are non-specific inhibitors that affect many other targets besides the NF-κB pathway. Plumbagin, a non-specific inhibitor, blocks NF-κB signaling and induces caspase-3 activity, thus decreasing cell viability and promoting apoptosis in TNBC cell lines [138]. Similarly, genistein, a major soy isoflavone, has anti-growth and pro-apoptotic effects in TNBC due to the inhibition of NF-κB activity via the Notch-1 pathway [139]. Specific anti-NF-κB strategies include targeting pathway constituents upstream of IKK, inhibiting IKK itself, inhibiting NF-κB activities in the nucleus or targeting NF-κB downstream effectors [137]. For example, dehydroxymethylepoxyquinomicin (DHMEQ), which inhibits nuclear translocation of NF-κB, reduced the activation of this pathway in TNBC cells leading to decreased growth and induction of apoptosis [140]. However, despite the abundance of information regarding the role of NF-κB in cancer and the substantial effort of researchers and the pharmaceutical industry, only a few inhibitors have found their way into the clinical practice in niche applications [137]. Clinical investigations of NF-κB mostly reported disappointing results due to the very high toxicity of systemic inhibitors and the pleiotropic effects of NF-κB [137]. Still, the data presented in this review reveals TNBC as one of the malignancies that could considerably benefit from NF-κB inhibition if the toxicity issues can be resolved.

#### 3.5.2. PTEN and PI3K-AKT-mTOR Pathway

PI3K-AKT-mTOR (PAM) pathway is one of the critical mechanisms by which cells control survival, growth, proliferation, and motility. Phosphoinositide 3-kinase (PI3K) transduces signals from growth factors and activates the AKT kinase [141]. Activation of AKT leads to phosphorylation of the mammalian target of rapamycin (mTOR), which, in turn, enhances protein synthesis and cell growth, giving malignant cells a significant advantage [141]. PAM activity is negatively regulated by the tumor suppressor phosphatase and tensin homolog (PTEN) [141]. 

PAM pathway is frequently hyperactivated in TNBC, chiefly due to PTEN loss, and is associated with adverse clinical course, aggressive tumors, and poor outcome [142,143]. PTEN loss also contributes to chemoresistance of breast cancer [144]. Similarly, highly expressed activated AKT was associated with chemoresistance in breast cancer [144] while mTOR inhibition sensitized resistant cells to cytotoxic agents [145]. In addition, AKT induces HIF-1, which, as we have seen, is a notable factor in chemoresistance [141]. Targeting the PAM pathway in conjunction with chemotherapy could be a useful strategy in aggressive TNBCs with PTEN loss. mTOR inhibitor, everolimus showed good activity against TNBC in preclinical investigations. Promising results were also obtained for NVP-BEZ235, a PI3K/mTOR inhibitor, in TNBC cell lines [146]. These findings have paved the way for clinical testing of PAM inhibitors. Alpelisib, is the first PI3K inhibitor to be approved for treatment of hormone receptor-positive, HER2 negative advanced or metastatic breast cancer harboring *PIK3CA* mutations [147]. A number of phase I and II clinical trials are underway investigating the effects of mTOR and PI3KA inhibitors, alone or in combination with chemotherapy mainly in advanced TNBC (NCT02531932, NCT01931163, NCT00761644, NCT01629615, NCT01623349, NCT01920061, NCT01884285, NCT02583542). Recently AKT has been revealed as a potent therapeutic target in the LOTUS trial [148]. A combination of AKT inhibitor ipatasertib with paclitaxel, prolonged progression-free and overall survival of TNBC patients compared to paclitaxel alone. Moreover, the benefit was greater in the *PIK3CA/AKT1/PTEN* altered population, highlighting the importance of careful patient selection. These findings imply that the PAM pathway might be critical for the maintenance and growth of a subset of TNBCs and have provided a rationale for the currently recruiting trial investigating ipatasertib in advanced TNBCs preselected for PIK3CA/AKT1/PTEN alterations (NCT03337724). Another AKT inhibitor, uprosertib, was recently evaluated in a phase II clinical trial on metastatic TNBC, but formal results are still pending (NCT01964924). AZD5363, also a novel AKT inhibitor, in combination with chemotherapy, showed a favorable effect on overall survival in a phase II trial on metastatic TNBC [149]. These results indicate that AKT might be a preferred PAM target in TNBC.

#### 3.5.3. JAK/STAT Pathway

JAK/STAT pathway consists of four proteins with Janus kinase domain (JAK1–3, TYK2) and seven proteins that comprise the signal transducer and activator of transcription protein family (STAT1–4, STAT5A, STAT5B, and STAT6). JAKs are cytoplasmic proteins associated with transmembrane receptors. Binding of an extracellular ligand (such as IL6, IL8) allows the trans-phosphorylation of JAKs which then phosphorylate STAT monomers. Activated STATs enter the nucleus and subsequently regulate the transcription of numerous target genes [150]. 

Abnormal JAK/STAT signaling has been implicated in numerous crucial malignant processes such as tumorigenesis, survival, proliferation, metastasis, immune suppression, angiogenesis, and anti-apoptosis [150]. Genetic profiling revealed a pro-inflammatory gene signature characteristic for TNBC. Among the identified genes, IL6 and IL8 were reported as crucial for TNBC growth both in vitro and in vivo while, simultaneously having little effect on ER+ cells [151]. Moreover, combined inhibition of IL6 and IL8 significantly reduced tumor growth, promoted apoptosis, and enhanced TNBC sensitivity to paclitaxel [151]. STAT3, a member of the JAK/STAT signaling pathway downstream from IL6/8, is highly expressed in TNBC and connected with tumor initiation, aggressive clinical behavior, unfavorable outcome, and resistance to chemotherapy [152,153]. STAT3 interacts and collaborates with NF-kB leading to chemoresistance in TNBC [154]. In addition, STAT3 was upregulated in TNBC stem cells resistant to doxorubicin [155]. STAT3 was also able to upregulate HIF1, thus proving its involvement in hypoxia-mediated chemoresistance in TNBC [156]. Interestingly, STAT3 upregulated the expression of ABC transporters further contributing to hypoxia-induced chemoresistance [156]. Since STAT3 has a strong oncogenic potential in TNBC; numerous inhibitors have been developed with diverse modes of action targeting STAT3 itself as well as its upstream regulators [157]. The research is predominantly in the preclinical stage of development, but the results regarding TNBC are encouraging [157]. STAT3 inhibitor, WP1066 was able to restore the sensitivity of TNBC cells to doxorubicin [158]. Moreover, simultaneous admission of STAT3 and HIF-1α inhibitors significantly increased the anti-tumor activity of cisplatin in TNBC under hypoxic conditions [159]. In a similar vein, the JAK2 gene was preferentially amplified in TNBC cells following chemotherapy treatment and its specific inhibitor, together with chemotherapy, was able to suppress tumor progression and growth [160]. According to Nascimento et al., several STATs and JAK1–2 were significantly upregulated in chemoresistant breast cancer cells [161]. The same authors also demonstrated that JAK inhibitor tofacitinib sensitized the cells to chemotherapy [161]. These promising preclinical results have inspired several clinical studies targeting STAT3 and JAK2 in various solid tumors [157]. JAK1/2 inhibitor ruxolitinib, in combination with neoadjuvant chemotherapy, is currently undergoing investigation in triple-negative inflammatory breast cancer (NCT02876302). A clinical trial evaluating STAT3 inhibitor TTI-101 in advanced cancers, including breast cancer, recently started recruiting participants (NCT03195699). AZD9150, a novel antisense nucleotide inhibitor of STAT3, is undergoing investigation in a phase I/II clinical trial together with durvalumab and paclitaxel in metastatic TNBC (NCT03742102).

#### 3.5.4. Receptor Tyrosine Kinases

PAM and JAK/STAT signaling pathways are used by numerous growth factors to bring about a multitude of biological outcomes. The upstream regulators of these pathways implicated in TNBC chemoresistance are EGFR and insulin-like growth factor-1 receptor (IGF-1R).

EGFR expression is markedly higher in TNBC compared to other breast cancer subtypes and has been reported in up to 64% of cases [162]. EGFR overexpression is even considered one of the hallmarks of TNBC. On the genetic level, *EGFR* gene amplification, but not mutations, correlates with protein expression [162]. These findings imply gene amplification as the most crucial mechanism behind increased EGFR expression in TNBC. In addition, several studies demonstrated that EGFR amplification and expression are connected with worse outcome in TNBC [162,163]. The EGFR pathway is also involved in the regulation of ABCG2 expression and function [164,165]. Moreover, EGFR inhibition resulted in the reversal of ABCG2-mediated chemoresistance in-vitro and in-xenograft models [166]. 

There are two basic strategies for targeting EGFR (and other receptor tyrosine kinases) in TNBC: the use of anticancer mAbs specific for the receptor and, second, the use of TKIs. Cetuximab, an anti-EGFR mAb, was investigated in metastatic TNBC together with cisplatin [167]. While the primary endpoint of the clinical trial (the overall response rate) was not achieved, combined treatment with cetuximab and cisplatin moderately increased progression-free and overall survival [167]. However, a phase II study of cetuximab in combination with carboplatin in metastatic TNBC was met with disappointing results [168]. Panitumumab, another EGFR mAb, showed mixed efficacy in clinical trials [169,170]. Interestingly, in a trial focusing on early, operable TNBC, the number of tumor-infiltrating lymphocytes was predictive of tumor response to panitumumab [171]. These findings imply an important connection between EGFR regulated pathways and the immune component of the tumor microenvironment. This has provided a rationale for the currently ongoing clinical trials of panitumumab in combination with chemotherapy in inflammatory TNBC (NCT02876107, NCT01036087). Another strategy to increase the effect of anti-EGFR mAbs is to use a combination of antibodies. Ferraro et al. showed a cooperative effect of anti-EGFR mAbs leading to enhanced inhibitory potential [172]. Despite initial success, several TKIs, such as gefitinib, afatinib, and lapatinib, showed disappointing results in clinical trials. The efforts to test existing and develop new TKIs are ongoing. For instance, a clinical trial investigating icotinib in metastatic TNBC is currently recruiting patients (NCT02362230). One proposed way to enhance the effect of EGFR inhibition is to combine anti-EGFR mAbs and TKIs, which seems to have a synergistic effect and could result in a stronger anti-tumor effect [173]. 

It is a puzzle why EGFR targeted therapy has poor performance in TNBC—a tumor characterized by high EGFR overexpression. One possibility is that mutations, not protein expression level, dictate the efficacy of anti-EGFR TKIs. One study identified two EGFR mutations in TNBC which are known as good predictors of TKI sensitivity in non-small cell lung cancer [174]. However, several other groups reported EGFR mutations as extremely rare events in TNBC with little clinical relevance [162,175]. The work of Ali and coworkers may provide an alternative answer to this puzzle in the form of what they termed the “EGFR paradox” [176]. According to the authors, the role of EGFR signaling changes during tumor progression akin to, for example, TGF-β. Namely, the examination of paired samples of primary tumors and their corresponding metastases revealed that EGFR was overexpressed in primary tumors while metastatic cells had markedly reduced EGFR expression and were intrinsically resistant to EGFR targeted therapy [176]. This phenomenon was observed in a number of human cancers, including TNBC [176]. The majority of recent and current clinical trials involving EGFR were performed on metastatic TNBC. Incidentally, the two clinical studies of panitumumab that reported the greatest benefit were conducted on operable, primary TNBC [169,171]. These findings imply that metastatic TNBC is not dependent on EGFR signaling for survival. Other growth factors and pathways compensate for the loss of EGFR signaling and their identification is urgently needed. It seems that a fundamental shift in our approach to EGFR targeted therapy in TNBC might be necessary. 

IGF-1R is a transmembrane receptor overexpressed in numerous human tumors. Insulin-like growth factors (IGFs) bind to the receptor activating the downstream signaling cascade, ultimately stimulating cell growth, proliferation, expression of ABC transporters, angiogenesis, and inhibiting apoptosis [177]. IGF-1R is expressed in up to 46% of TNBCs and is associated with poor survival [178]. IGF-1 was demonstrated to interact with the Wnt/β-catenin pathway and was expressed at a higher level in CSCs compared to bulk tumor cells in TNBC [84]. Importantly, β-catenin inhibition markedly reduced IGF-1 levels, which decreased the self-renewal and growth abilities of CSCs [84]. Moreover, IGF-1R overexpression is connected with chemoresistance in various cancers [177]. IGF-1R expression was upregulated in breast tumors following neoadjuvant chemotherapy and was associated with shorter overall survival [179]. Studies on TNBC cell lines and primary tumor xenografts have shown good efficacy of IGF-1R inhibitors [180]. Promising preclinical results in regard to IGF-1R inhibitors encouraged many subsequent clinical trials in various settings, including breast cancer. Unfortunately, the results were generally disappointing [181]. Recent efforts are mostly focusing on anti-IGF-1R mAbs in combination with chemotherapy or other signaling disruptors [181]. Another proposed strategy is the neutralization of ligands, not receptors, by mAbs. Two of these, MEDI-573 and BI836845, are currently being investigated [182].

### 3.6. MicroRNAs

MicroRNAs are small, non-coding, single-stranded RNAs that regulate crucial biological processes at the posttranscriptional level by repressing the translation of proteins. MicroRNAs can act as both tumor suppressors and oncogenes, depending on which proteins are repressed. Distinctive microRNA expression profiles/signatures are characteristic of specific diseases. In recent years, a specific cluster of microRNAs has been identified in TNBC and linked with tumor invasiveness, EMT, stemness, migration, metastasis, and chemoresistance. For instance, miR-20a-5p was highly expressed in TNBC tissues and cell lines, promoted migration and invasiveness of TNBC cells while, conversely, its depletion had strong opposite effects [183]. This result is paralleled by miR-224, which exhibited a significantly higher level of expression in TNBC compared to luminal breast cancer. Overexpression of miR-224 had a strong negative effect on caspase-9, which lead to the inactivation of the apoptotic pathway and increased the viability, migration, and invasiveness of TNBC cells [184]. Similar findings were reported for miR-221/222 that was dramatically overexpressed in TNBC cell lines and tissues where it promoted cellular survival, proliferation, EMT, and migration via the Wnt/β-catenin pathway [185]. A number of other microRNAs were abnormally expressed in TNBC and connected with disease outcome. Increased expression levels of miR-21, miR-210, miR-454, and miR-27a/b were associated with poor survival of TNBC patients. At the same time, the reduction of tumor suppressor miR-155 expression was predictive of shorter survival [186]. 

Accumulating evidence suggests that microRNAs have an important role in both TNBC resistance and sensitivity to cytotoxic drugs. Song et al. demonstrated that miR-301b was upregulated in TNBC cell lines where it inhibited 5-fluorouracil induced apoptosis [187]. MiR-105 and miR-93-3p were shown to induce cisplatin chemoresistance, stemness, and metastasis in TNBC trough Wnt/β-catenin signaling [188]. Overexpression of miR-620 promoted gemcitabine resistance of TNBC cells [189]. The up-/downregulation of an entire cluster of microRNAs was found to influence doxorubicin resistance in TNBC [190]. On the other hand, several microRNAs were shown to enhance the chemosensitivity of TNBC cells to various cytotoxic agents such as paclitaxel and doxorubicin [191,192]. Moreover, it seems that specific microRNA signatures are associated with pCR in TNBC [193].

Because of their wide-ranging effects, microRNAs represent a promising novel target in cancer therapy. Two basic strategies have been developed: the inhibition of oncogenic microRNAs and rehabilitation of tumor suppressor microRNAs function using substitutes [194]. Various therapeutic molecules are deployed in realizing these strategies. For the inhibition of oncogenic microRNAs, the following agents are used: anti-microRNA oligonucleotides, microRNA sponges, small RNA zipper molecules, antagomiRNAs, locked nucleic acid anti-miRNAs, and small molecule inhibitors. For instance, antisense-microRNA-21 and antisense-microRNA-10b co-delivery using nanoparticles significantly reduced TNBC cell proliferation and tumor growth in murine TNBC models [195]. MicroRNAs mimics and microRNAs coded by expression vectors are utilized for the restoration of tumor suppressor microRNAs [194]. Hashemi et al. reported that the restoration of miR-193b-3p and miR-31-5p expression levels using miR-mimic recombinant vectors dramatically reduced the migration and invasion of TNBC cells [196]. 

MicroRNAs are currently being vigorously investigated as potential biomarkers in various human cancers. Indeed, the use of microRNAs as biomarkers is nearing implementation in the clinic. On the other hand, clinical data on microRNAs as therapeutics in solid tumors is scant as this field is still in its infancy. MicroRNAs-based therapeutic approach holds much potential but will require improvements in delivery systems, toxicity, selectivity, and specificity if it is to find its way into the daily clinic. When these issues are resolved, microRNAs will undoubtedly become crucial targets for TNBC treatment.

### 3.7. TNBC Heterogeneity

Another obstacle standing in the way of successful chemotherapy treatment of TNBC is its high heterogeneity. TNBC is not a single disease but an umbrella term encompassing several entities with significant differences in prognosis, patterns of relapse, and response to chemotherapy. In a landmark study, Lehmann et al. conducted a detailed dissection of TNBC gene expression profiles showing that these tumors can be stratified into six subtypes [146]. The authors later refined these subtypes into four: basal-like 1 (BL1), basal-like 2 (BL2), mesenchymal (MES) and luminal androgen receptor (LAR) [197]. However, the molecular profiling of TNBC is an ongoing effort. Other studies have reported similar but different definitions of TNBC subtypes [198,199]. Nevertheless, LAR, BL, and MES molecular profiles are consistently present. These subtypes display varying levels of chemoresistance, which is reflected in their pCR rates [200]. LAR appears to be the most resistant subtype based on the information received from several clinical trials and preclinical studies [200,201]. Interestingly, Ashgar and colleagues showed that LAR tumors are relatively quiescent, which could partially explain their chemoresistance [202]. As the name implies, LAR tumors are characterized by the androgen receptor (AR) expression and luminal genetic profiles. In keeping with luminal properties, *PI3KCA* mutations are much more frequent in LAR tumors compared to other TNBC subtypes [203]. As we have already discussed, the PAM pathway is strongly implicated in chemoresistance and could represent a suitable approach for targeting LAR in addition to AR antagonists. After LAR, the lowest pCR rates were observed in MES tumors [200]. Moreover, TNBC cell cultures with mesenchymal properties, like MDA-MB-231 and hs578t, display high levels of chemoresistance. The MES subtype of TNBC is enriched in gene expression signatures linked with EMT and stemness [146]. In Section 3.2 of this review, we have extensively documented the crucial role of CSCs and the associated developmental pathways in TNBC chemoresistance. Therefore, therapies targeting these pathways may be most effective in treating the MES subtype of TNBC. On the other end of the spectrum is the BL group, which demonstrates high pCR rates and is the most predominant TNBC subtype [200]. This group of TNBCs is characterized by robust proliferation and is enriched in genes involved in cell cycle and DNA damage response [146]. *BRCA1/2* is frequently inactivated in BL1 subtype due to mutations or hypermethylation. This leads to deficiencies in DNA damage repair, thus making these tumors more susceptible to DNA damaging agents [146]. Indeed, platinum-based chemotherapy significantly increased the pCR rates of TNBC patients that harbor germ-line *BRCA* mutations [204]. Poly (ADP-ribose) polymerase (PARP) inhibitors also demonstrated good activity in this TNBC subtype owning to synthetic lethal effect in combination with *BRCA1* inactivation. Recently, PARP inhibitors, olaparib and talazoparib were approved for use in HER2 negative, *BRCA* mutated, metastatic breast cancer pretreated with chemotherapy [205,206]. Finally, in their original study, Lehmann et al. also identified the so-called, immunomodulatory TNBC subtype [146]. This gene expression profile was later proven to originate from tumor-infiltrating lymphocytes (TILs), and not from the TNBC itself [197]. Therefore, IM phenotype is not a distinct subtype itself, and its signature can overlap with the established molecular subtypes. Still, the presence of TILs is a favorable prognostic marker, especially if observed in the residual tumor following neoadjuvant chemotherapy [207]. These findings have provided a rationale for the testing of immune-checkpoint inhibitors in TNBC. Atezolizumab, a mAb against programmed cell death-ligand 1 (PD-L1), in combination with nab-paclitaxel, demonstrated good efficacy in TNBC [208]. It prolonged both progression-free and overall survival of patients, and the effect was especially pronounced in patients whose tumors expressed PD-L1 [208]. Based on these results, atezolizumab was recently approved for use for women with advanced or metastatic TNBC expressing PD-L1 [209]. TNBC seems to be a spectrum of several diseases with overlapping genetic alterations. Thus, devising therapeutic strategies tailored to specific TNBC subtypes is a challenging but worthwhile approach. As illustrated in this review, TNBC subtypes have distinct sensitivities to standard cytotoxic agents and as such, should be considered when deciding on the therapeutic management of TNBC. The characteristics of TNBC subtypes and proposed tailored approaches in treatment are summarized in Table 1.

## 4. Conclusions and Future Directions

TNBC is the most aggressive subtype of breast cancer characterized by poor survival and a high incidence of distant metastases. Limited treatment options are a major contributing factor to the poor outcome of TNBC. Standard chemotherapy remains the backbone of TNBC systemic treatment; however, these tumors often become resistant to cytotoxic drugs. There is an urgent medical need to elucidate the molecular drivers behind TNBC chemoresistance. This goal has been aggressively pursued in the last decade. The ensuing research has dramatically advanced our understanding of the elaborate mechanisms that govern TNBC resistance to chemotherapy. The present review highlights the complexity of TNBC chemoresistance, which is induced by the interaction and collaboration of numerous factors and signaling pathways. Completely untangling this network remains a significant challenge but is vital for the identification of new treatment targets. Data generated so far has spurred the development of a multitude of therapeutic agents with the aim of overcoming resistance in TNBC. This multipronged approach includes neutralizing ABC transporters; targeting developmental pathways and breast CSCs; exploiting tumor hypoxia, stopping tumors from circumventing apoptosis, inhibiting signaling pathways with critical roles in TNBC survival and confronting TNBC heterogeneity with subtype-specific treatment modalities. All of the strategies highlighted in the present review represent promising opportunities to sensitize TNBCs to the current standard of care chemotherapy. However, despite the immense effort, only a few of these agents have made the transition from research to clinical practice. Toxicity, specificity, and selectivity are overarching concerns in all approaches. Another issue shared among all of the emerging strategies is the selection of patients that would benefit the most from the given agent. A single pathway may not be crucial for cell survival in all TNBCs or all stages of progression. Indeed, several clinical trials have demonstrated the value of pre-selecting patients based on specific biomarkers [132,148]. By evaluating an inhibitor of a specific pathway in the whole TNBC population, its efficacy in the subgroup, depending on the pathway in question, may be masked. Thus, the development of a promising agent might be discontinued, and patients robed of potential treatment. The necessity of validating and incorporating biomarkers in future clinical studies is evident. Another question is how these novel therapeutics should be implemented, whether as single agents or combined treatment. While some of the agents outlined in this review showed good efficacy as monotherapy, extensive TNBC heterogeneity and crosstalk between signaling pathways will likely require combination therapy if treatment is to be successful. Most recent clinical studies on TNBC that demonstrated improvements in patient benefit used the combination treatment strategies, especially combinations of specific agents and standard chemotherapy (NCT02876302, NCT02672475, NCT02694224). Since these inhibitors target different mechanisms, they often show good synergistic effects and acceptable toxicity profiles when used together with cytotoxic drugs. Going forward, it is clear that future clinical studies in TNBC will need to focus on biomarker integration, precise patient stratification, and novel combinatorial regimens tailored to be effective in different patient subgroups.

## Figures and Tables

**Table 1:** Classification of TNBC subtypes and potential therapeutic strategies—recent clinical trials.

TNBC Subtype and Its Characteristics [147,198]	Promising Therapy	Clinical Studies
**Basal-like 1** **Cell cycle, cell division, DNA damage response.** **High pCR rate**	Platinum	**NCT02441933**, Phase III, Recruiting, Carboplatin in combination with standard chemotherapy in early TNBC
		**NCT01881230**, phase II/III, superior efficacy of Nab Paclitaxel/Carboplatin combination in metastatic TNBC
	PARP inhibitors	**NCT02000622**, Phase III, Olaparib as monotherapy significantly increased PFS and reduced the risk of progression and death in metastatic breast cancer including TNBC
		**NCT01945775**, Phase III, Talazoparib as monotherapy significantly increased PFS in advanced breast cancer including TNBC
	Aurora inhibitors	**NCT01639248**, Phase II, ENMD-2076 in previously treated, advanced or metastatic TNBC
		**NCT00858377**, Phase I, AMG 900 in taxane-resistant TNBC failed to provide clinical benefit
	CHK1 inhibitors	**NCT01359696**, Phase I, GDC-0425 in combination with gemcitabine showed preliminary anti-tumor activity in *TP53*-mutated TNBC
		**NCT00779584**, phase I, MK-8776 demonstrated preliminary clinical efficacy with acceptable toxicity in advanced solid tumors
**Basal-like 2** **Growth factor signaling, myoepithelial markers, activated glycolysis and gluconeogenesis pathways.** **Moderate pCR rate** **Shares susceptibility to platinum and PARP inhibitors with BL1**	EGFR pathway inhibitors	**NCT00463788**, Phase II, Cetuximab in combination with cisplatin increased PFS and OS in metastatic TNBC
		**NCT01036087**, Phase II, Panitumumab in combination with nab-paclitaxel and carboplatin demonstrated highest ever reported pCR rates in inflammatory TNBC
		**NCT02362230**, Phase II, Recruiting, Icotinib is being evaluated in previously treated metastatic TNBC
	IGF1R pathway inhibitors	**NCT01340040**, Phase I, MEDI-573 was well tolerated but showed limited activity as monotherapy in advanced solid tumors
		**NCT00626106**, Phase II, Ganitumab in combination with endocrine therapy failed to improve outcome in patients with previously treated HR+ advanced or metastatic breast cancer
**Mesenchymal** **EMT, stemness, growth factor signaling, angiogenesis, high rate of PAM pathway aberrations.** **Low pCR rates**	TGF-β inhibitors	**NCT02672475**, Phase I, Recruiting, Galunisertib in combination with paclitaxel in metastatic TNBC
	Notch inhibitors	**NCT01876251**, Phase I, PF-03084014 in combination with docetaxel was well tolerated and showed clinical benefit in patients with advanced TNBC
		**NCT00645333**, phase I/II, MK-0752 together with docetaxel had limited anti-tumor activity but reduced breast CSC burden in advanced or metastatic breast cancer
	Wnt/β-catenin inhibitors	**NCT01351103**, Phase I, Recruiting, LGK974 in patients with Wnt-ligand dependent malignancies including TNBC
	Hedgehog inhibitors	**NCT02027376**, Phase I, Sonidegib in combination with docetaxel was well tolerated and showed preliminary anti-tumor activity in advanced, pre-treated TNBC
		**NCT02694224**, Phase II, Recruiting, Vismodegib in combination with standard neoadjuvant chemotherapy in TNBC
	PI3K inhibitors	**NCT01629615**, Phase II, BKM120 as monotherapy in metastatic TNBC
		**NCT01884285**, Phase I, AZD8186 as monotherapy or in combination with other agents in advanced solid tumors including TNBC, demonstrated preliminary anti-tumor activity with serious adverse events
	mTOR inhibitors	**NCT02531932**, Phase II, Recruiting, Everolimus together with carboplatin in advanced TNBC
		**NCT01920061**, Phase I, Recruiting, Gedatolisib in combination with either cisplatin or docetaxel in TNBC
	AKT inhibitors	**NCT02162719**, Phase II, Ipatasertib in combination with paclitaxel, increased PFS and OS of patients with metastatic TNBC compared to paclitaxel alone
		**NCT02423603**, Phase II, AZD5363 in combination with paclitaxel, prolonged the PFS and OS of patients with metastatic TNBC compared to paclitaxel alone
	Anti-angiogenic therapy	**NCT01176669**, Phase II, Apatinib as a single agent demonstrated clinical benefit in pre-treated metastatic TNBC
		**NCT03348098**, Phase II, Recruiting, Apatinib in combination with paclitaxel as neoadjuvant treatment for locally advanced TNBC
		**NCT01234337**, Phase III, Sorafenib in combination with capecitabine failed to show clinical benefit compared to capecitabine alone in advanced or metastatic HER2-negative breast cancer including TNBC
**Luminal androgen receptor** **Hormonal-mediated signaling, androgen receptor, *PI3KCA* mutations.** **Very low pCR rate**	Androgen receptor	**NCT03055312**, Phase III, Recruiting, Bicalutamide as single agent compared to the efficacy of standard chemotherapy in metastatic, AR+ TNBC
		**NCT01889238**, Phase II, Enzalutamide as monotherapy was well tolerated and showed clinical benefit in patients with advanced AR+ pre-treated TNBC
	PI3K inhibitors	**NCT02457910**, Phase I/II, Enzalutamide in combination with Taselisib (PI3K inhibitor) in patients with AR+, metastatic TNBC
	Heat shock protein 90	**NCT01677455**, Phase II, Ganetespib as a single agent in TNBC patients who were not subjected to prior systemic treatment in the metastatic setting
		**NCT02474173**, Phase I, Recruiting, Onalespib in combination with paclitaxel in patients with metastatic TNBC
**Immunomodulatory** **Immune-mediated signaling**	Checkpoint inhibitors	**NCT02425891**, Phase III, Atezolizumab in combination with nab–paclitaxel prolonged both PFS and OS of patients with metastatic TNBC, especially in patients with PD-L1 expressing tumors
		**NCT02926196**, Phase III, Recruiting, Avelumab as adjuvant treatment for high-risk TNBC
	JAK/STAT pathway	**NCT02876302**, Phase II, Recruiting, Ruxolitinib, in combination with standard neoadjuvant chemotherapy in triple-negative inflammatory breast cancer
		**NCT03195699**, Phase I, Recruiting, TTI-101 as monotherapy in advanced cancers including breast cancer

PFS—progression free survival, OS—overall survival, AR+—androgen receptor positive. Details of the presented trials can be obtained by searching the trial identifier number in the US National Institutes of Health Registry (https://clinicaltrials.gov/).
